# Intimate Partner Violence and Electronic Health Interventions: Systematic Review and Meta-Analysis of Randomized Trials

**DOI:** 10.2196/22361

**Published:** 2020-12-11

**Authors:** Ditte S Linde, Aleksandra Bakiewicz, Anne Katrine Normann, Nina Beck Hansen, Andreas Lundh, Vibeke Rasch

**Affiliations:** 1 Department of Clinical Research University of Southern Denmark Odense Denmark; 2 Department of Gynaecology & Obstetrics Odense University Hospital Odense Denmark; 3 Department of Public Health University of Southern Denmark Esbjerg Denmark; 4 Odense Patient Data Explorative Network Odense University Hospital Odense Denmark; 5 Department of Psychology University of Southern Denmark Odense Denmark; 6 Centre for Evidence-Based Medicine Odense Odense University Hospital Odense Denmark; 7 Department of Infectious Diseases Hvidovre Hospital Copenhagen Denmark

**Keywords:** eHealth, randomized trials, intimate partner violence, domestic violence, abuse, depression, PTSD

## Abstract

**Background:**

Intimate partner violence (IPV) is a major public health concern. eHealth interventions may reduce exposure to violence and health-related consequences as the technology provides a safe and flexible space for the target population. However, the evidence is unclear.

**Objective:**

The goal of the review is to examine the effect of eHealth interventions compared with standard care on reducing IPV, depression, and posttraumatic stress disorder (PTSD) among women exposed to IPV.

**Methods:**

We searched EMBASE, MEDLINE, Cochrane Central Register of Controlled Trials, PsycInfo, Scopus, Global Health Library, ClinicalTrials.gov, and International Clinical Trials Registry Platform for published and unpublished trials from inception until April 2019. Trials with an eHealth intervention targeting women exposed to violence were included. We assessed risk of bias using the Cochrane Risk of Bias Tool. Trials that reported effect estimates on overall IPV; physical, sexual, and psychological violence; depression; or posttraumatic stress disorder were included in meta-analyses.

**Results:**

A total of 14 trials were included in the review; 8 published trials, 3 unpublished trials and 3 ongoing trials. Of the 8 published trials, 2 were judged as overall low risk of bias trials. The trials reported 23 types of outcomes, and 7 of the trials had outcomes that were eligible for meta-analyses. Our pooled analyses found no effect of eHealth interventions on any of our prespecified outcomes: overall IPV (SMD –0.01; 95% CI –0.11 to 0.08; *I*^2^=0%; 5 trials, 1668 women); physical violence (SMD 0.01; 95% CI –0.22 to 0.24; *I*^2^=58%; 4 trials, 1128 women); psychological violence (SMD 0.07; 95% CI –0.12 to 0.25; *I*^2^=40%; 4 trials, 1129 women); sexual violence (MD 0.36; 95% CI –0.18 to 0.91; *I*^2^=0%; 2 trials, 1029 women); depression (SMD –0.13; 95% CI –0.37 to 0.11; *I*^2^=78%; 5 trials, 1600 women); and PTSD (MD –0.11; 95% CI –1.04 to 0.82; *I*^2^=0%; 5 trials, 1267 women).

**Conclusions:**

There is no evidence from randomized trials of a beneficial effect of eHealth interventions on IPV. More high-quality trials are needed, and we recommend harmonizing outcome reporting in IPV trials by establishing core outcome sets.

**Trial Registration:**

PROSPERO International Prospective Register of Systematic Reviews CRD42019130124; https://www.crd.york.ac.uk/prospero/display_record.php?RecordID=130124

## Introduction

### Background

Intimate partner violence (IPV) is defined as “a behavior by an intimate partner or ex-partner that causes physical, sexual, or psychological harm, including physical aggression, sexual coercion, psychological abuse, and controlling behaviors” [1]. It is also known as domestic abuse, domestic violence, or battering, and it is a major public health issue and a violation of human rights [2,3]. IPV can affect both men and women, yet most survivors are women [4,5]. Research on the prevalence of male survivors of IPV is scarce, and to the best of our knowledge, there is currently no global estimate on the magnitude of problem. However, a 2015 national survey from the United States reported that 11% of American men experienced some form of IPV during their lifetime [6]. Globally, approximately 1 in 3 women will experience physical or sexual violence from their partner during their lifetime. However, there are regional differences with the highest prevalence being found in Southeast Asia, the Eastern Mediterranean region, and Africa (around 37%) while the lowest prevalence is found in high-income countries (around 23%) [7].

IPV can have a number of immediate and long-term health consequences including physical injury, depression, anxiety, posttraumatic stress disorder (PTSD), suicidality, and substance abuse as well as gastrointestinal and gynecologic problems [7,8]. The worst cases can lead to homicide [9]. Further, the fetuses or children of the IPV survivors may be indirectly exposed to IPV, which can result in induced abortion, preterm birth, low birth weight, and infant mortality as well as developmental and behavioral problems later in life [10]. Often people experiencing IPV do not report the violence or delay seeking counseling due to a number of barriers, including stigma, embarrassment, and fear of the perpetrator [2].

eHealth is defined as the use of information and communication technologies for health [11]. It is a diverse concept that encompasses the subareas mobile health (mHealth) and telehealth [12]. It has been hypothesized that eHealth interventions have potential to reduce IPV exposure and its health-related consequences as the technology provides a safe and flexible space for the target population compared with traditional face-to-face approaches [13]. However, evidence of the effect of eHealth on IPV is unclear. Two Cochrane reviews from 2014 and 2015 assessed interventions for prevention and reduction of IPV among pregnant women [14] and women in general [15]. Some eHealth interventions were included in these reviews and showed mixed results [16-18]. New trials have since been published, and to our knowledge there is no systematic review specifically addressing eHealth interventions and their effect on reducing IPV and IPV-related health consequences.

### Objectives

The goal of the review is to estimate the effect of eHealth interventions compared with standard care on reducing overall IPV (physical, sexual, or psychological violence), type-specific IPV, depression, and PTSD among women exposed to IPV.

## Methods

### Protocol and Registration

The protocol was registered at the International Prospective Register for Systematic Reviews (PROSPERO) prior to study conduct [CRD42019130124] (registration date: April 15, 2019) [19]. The review is reported according to the Preferred Reporting Items for Systematic Reviews and Meta-Analyses (PRISMA) 2009 checklist [20] (Multimedia Appendix 1).

### Eligibility Criteria

We included published and unpublished randomized controlled trials, including pilot trials, in any language and setting. Further, we included trials of women exposed to any type of IPV by a current or former partner at any point in life. All types of eHealth interventions (eg, videos, text messages or social media interventions) were included and eHealth interventions had to be compared with standard of care, placebo-like interventions (eg, online counseling on another health issue than IPV), other eHealth interventions, or another type of interventions (eg, face-to-face counseling). We excluded trials of survivors of other forms of violence (eg, dating violence or gang violence), trials restricted to survivors of IPV with substance problems or sexual minorities, and trials targeting both men and women if separate data for women were not available.

### Information Sources and Search Strategy

We searched EMBASE, MEDLINE, PsycInfo, Scopus, Cochrane Central Register of Controlled Trials, and Global Health Library for trials from inception up to April 2019 (Multimedia Appendix 2). The search strategy was developed in collaboration with an experienced research librarian. In addition, we searched reference lists of included trials, the International Clinical Trials Registry Platform, and ClinicalTrials.gov in June 2019 for unpublished or ongoing trials.

### Outcomes

Our primary outcome was overall IPV (physical and/or sexual and/or psychological violence). Our secondary outcomes were type-specific IPV (ie, physical violence, psychological violence, and sexual violence), depression, and PTSD.

### Study Selection

After removing duplicates, two authors (AB, AKNN) screened titles and abstracts for obvious exclusion and assessed full-text papers using the web-based systematic review production tool Covidence [21]. Disagreements were resolved through discussion, and there was no need for involvement of an arbiter.

### Data Extraction

Two authors (AB, AKNN) identified relevant outcomes, and one author (AB) extracted data verbatim into a standardized Excel (Microsoft Corp) template. One author (DSL) extracted outcome data for meta-analysis and verified the other data. Extracted data included first author, publication year, title, journal name, registry record ID, length of study, country, setting, objective, eligibility criteria, number of participants, number of males and females, mean age, description of interventions, primary and secondary outcomes, and funding source. Corresponding authors were contacted for unpublished data.

### Risk of Bias Assessment

Two authors (AB, AKNN) independently assessed published trials for risk of bias using the Cochrane Risk of Bias Tool [22]. The following domains were assessed: sequence generation and allocation concealment (selection bias), blinding of participants and personnel (performance bias), blinding of outcome assessors (detection bias), incomplete outcome data (attrition bias), and selective outcome reporting (reporting bias). Domains were assessed as having low risk, high risk, or unclear risk of bias. Trials were judged as overall low risk of bias if they had low risk of selection bias, detection bias, and reporting bias. All other trials were judged as having high risk of bias. Disagreements were resolved through discussion. In case of disagreements, a third coauthor (DSL) made a final decision.

### Data Analysis

For our descriptive analysis of study outcomes and outcome measurements scales, we constructed a multiple outcome matrix using the methodology developed by Mayo-Wilson and colleagues [23]. Meta-analyses were conducted on reduction of IPV (overall or physical, sexual, or psychological violence), PTSD, and depression. Meta-analyses were done using RevMan 5.3 (Cochrane). We planned to use both continuous and dichotomous outcome data, but no trials reported dichotomous outcome data. As we expected trials to be heterogeneous in terms of methodology, types of populations, and interventions, we used random effects models and the inverse-variance method. If trials reported continuous data using the same outcome measure (ie, similar scale), we analyzed data using mean difference, and if trials used different scales, we analyzed data using standardized mean difference and calculated corresponding 95% confidence intervals. We assessed statistical heterogeneity by using *I*^2^. If trials had several time points for follow-up, we used the latest time point in our analyses. We conducted subgroup analyses comparing overall low risk of bias trials with high risk of bias trials, type of eHealth intervention, and type of scale for our primary outcome.

## Results

### Summary

**Figure 1:**
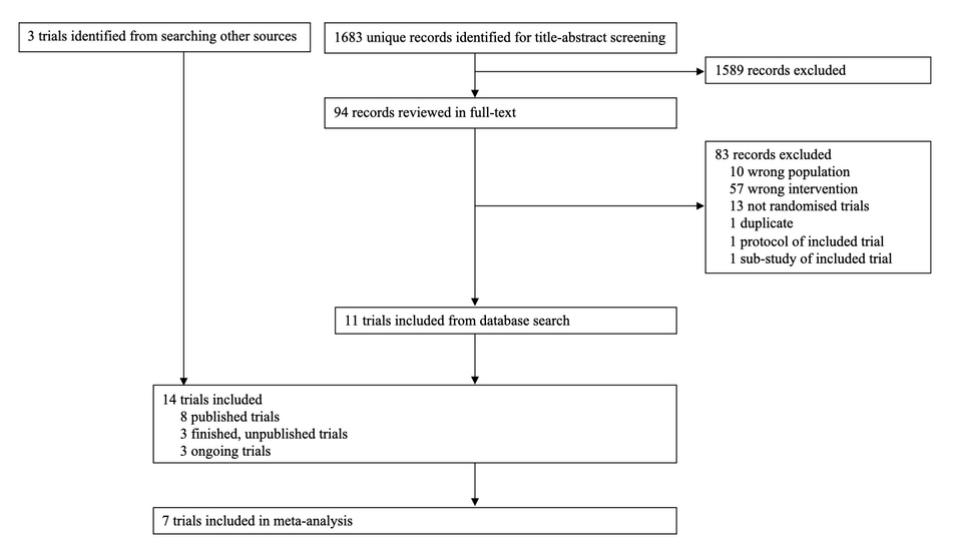
Preferred Reporting Items for Systematic Reviews and Meta-Analyses (PRISMA) flowchart.

We identified 1683 unique records, and excluded 1589 records after screening titles and abstracts (Figure 1). Of the 94 records reviewed in full text, 83 were excluded, leaving 11 trials for inclusion [8,24-36]. Three additional trials were included from searching other sources, leading to the inclusion of 14 trials in the review. Of the 14 trials, 8 were finished and published [8,24-31], 3 were finished but unpublished [32,35,37], and 3 were ongoing [33,34,36] (Table 1). Nine corresponding authors were contacted for clarification of data or unpublished data [8,28,29,32-35,37,38]. Seven authors replied [28,29,32-35,37], and 2 provided unpublished data [29,35] in the form of a different standard deviation, which was used in the meta-analysis [29], and tabulated data for a finished trial in the form of a draft manuscript [35]. However, as we were unable to resolve queries concerning the data, we decided not to include the data in our review.

**Figure 2:**
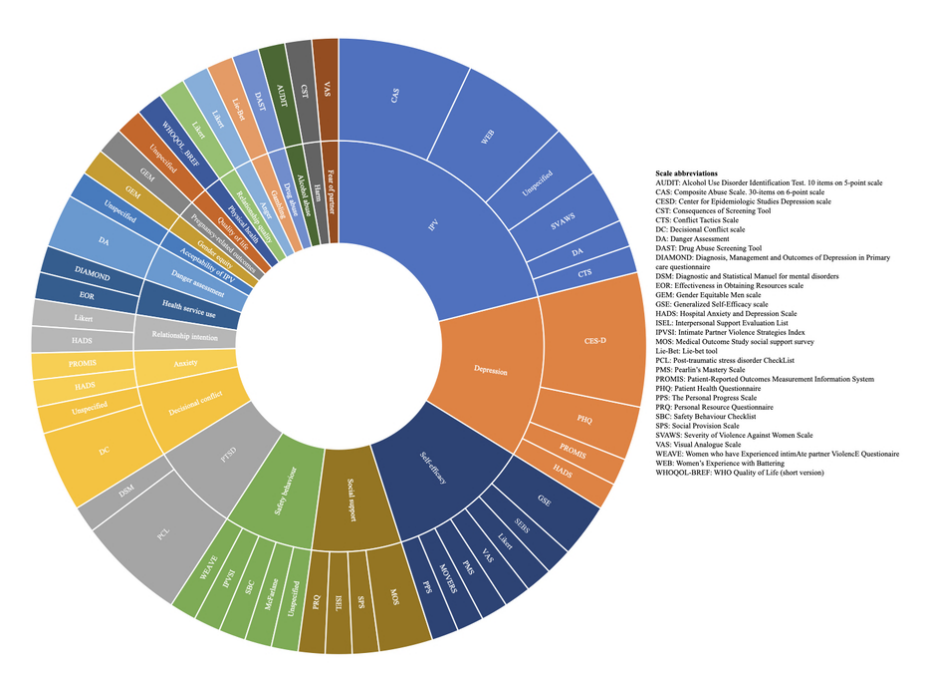
Outcomes and outcome measurement scales in trials with eHealth interventions and intimate partner violence.

The 8 trials were published from 2002 to 2019 enrolling 2147 women in total (median 202 participants per trial; Table 1). In the 6 trials that were either unpublished or ongoing, 3966 women were planned to be enrolled (median 450 participants per trial). The published trials were conducted in the United States (n=6), Australia (n=1), and New Zealand (n=1) and, except for 1 study that targeted couples [31], solely included women. The mean age of the participants ranged from 27.6 to 40.0 years, and follow-up varied from 1.5 to 12 months. Recruitment strategies varied across trials from general advertisements on television or online spaces to more specific advertisement in family court waiting areas and health clinics. All trials were 2-arm except for one 3-arm trial [28]. Three trials compared an online safety decision aid with a control website or standard safety planning [8,24,25], 1 trial compared online education on IPV with online popular TV shows [27], 2 trials assessed telephone support compared with standard care [29,30], 1 trial compared email modules to placebo email modules [31], and the 3-armed trial compared email modules to standard care or face-to-face modules [28]. Types of outcomes and how they were measured differed greatly across the 14 trials; 23 (median 4; interquartile range 3.75) types of outcomes and 49 outcome measurements were reported (Figure 2). For example, 7 different scales were used to measure self-efficacy, 5 different scales were used to measure overall IPV, and 4 different scales were used to measure depression.

**Table 1:** Characteristics of trials included in the review.

Trial type		Country	Trial size (n)	Women, %	Age in years, mean	Follow-up (months)	Recruitment	Intervention^a^	Comparator	Primary outcome measure (scale)
**Finished, published**										
	Hegarty [24]	Australia	422	100	33.7	12	Online advertisement; compensation for time up to Aus $150 (US $110)	Online safety decision aid	Control website	Self-efficacy (GSE^b^)
	Koziol-McLain [25]	New Zealand	412	100	29.0 (median)	12	TV advertisements and flyers at health clinics	Online safety decision aid	Control website	Depression (CES-D^c^)
	Zlotnick [27]	US	53	100	27.6	3	Pregnant women seeking mental health care who screened positive for IPV^d^	Online education on IPV	Online popular TV shows	Satisfaction with intervention (CSQ-8-R^e^)
	Glass [8]	US	721	100	33.4	12	Online advertisement, flyers at health clinics and public toilets	Online safety decision aid	Control website	IPV (SVAWS^f^, WEB^g^)
	Constantino [28]	US	32	100	40	1.5	Family court waiting areas, legal services, women’s shelters	email modules with IPV support (arm 1) or face-to-face modules with IPV support (arm 2)	Standard care	Anxiety (PROMIS^h^)
	Stevens [29]	US	253	100	29.2	6	Women at pediatric emergency departments who screened positive for IPV	Telephone support	Standard care	IPV (CAS^i^; WEB)
	Braithwaite [31]	US	104	50	32.4	12	Online, posters, and newspaper advertisements	Emails, modules with relationship communication skills, and problem-solving training	Placebo emails; modules with information about depression, anxiety, and healthy relationships	Physical and psychological violence (CTS^j^)
	McFarlane [30]	US	150	100	30.3	6	Family violence unit	Telephone support	Standard care	Safety behavior
**Finished, unpublished**										
	Clark [35] (NCT02942433, retrospectively registered)	Nepal	1440^k^ (36 clusters)	50	—	18	Women participating in survey at development centers	Weekly radio drama, SMS^l^, phone calls, and discussion groups	Weekly radio drama, SMS	Physical and/or sexual violence (unspecified)
	Ford-Gilboe [32]	Canada	450	100	—	12	Advertisements in various online spaces	Online safety decision aid	General online safety information	Depression (CES-D)
	PACTR201804003321122 [37]	Kenya	450	100	—	3	Study centers in Nairobi settlement	App with safety decision aid	Standard care	Sexual and reproductive coercion (unspecified)
**Ongoing**										
	Henriksen [34] (NCT03397277)	Norway	525	100	—	3	Women attending antenatal clinics who screened positive for IPV	Safety decision aid video	Control video	Safety behavior (McFarlane’s list)
	Sabri [33] (NCT03265847)	US	1250	100	—	12	Written/verbal invitation to indigenous, immigrant, and refugee women; invitations sent through list servers, emails, and snowballing	Online and app safety decision aid	Control website	Physical violence (CTS-2)
	NTR7313 [36]	Netherlands	198	100	—	6	Women self-identifying as IPV survivors through questions and registration online for SAFE (eHealth intervention)	Online safety decision aid	Not reported	Self-efficacy (GSE)

^a^Study with 2 intervention arms is specified by arm 1 and arm 2. Other trials had 1 intervention arm that could consist of multiple elements.

^b^GSE: General Self-Efficacy Scale.

^c^CESD: Center for Epidemiologic Studies Depression Scale.

^d^IPV: intimate partner violence.

^e^CSQ-8-R: Client Satisfaction Questionnaire, Revised–8 item.

^f^SVAWS: Severity of Violence Against Women Scale.

^g^WEB: Women’s Experience With Battering Scale.

^h^PROMIS: Patient-Reported Outcomes Measurement Information System.

^i^CAS: Composite Abuse Scale.

^j^CTS: Conflict Tactics Scale.

^k^Unpublished data reported by corresponding author.

^l^SMS: short message service (text messaging).

### Risk of Bias

**Figure 3:**
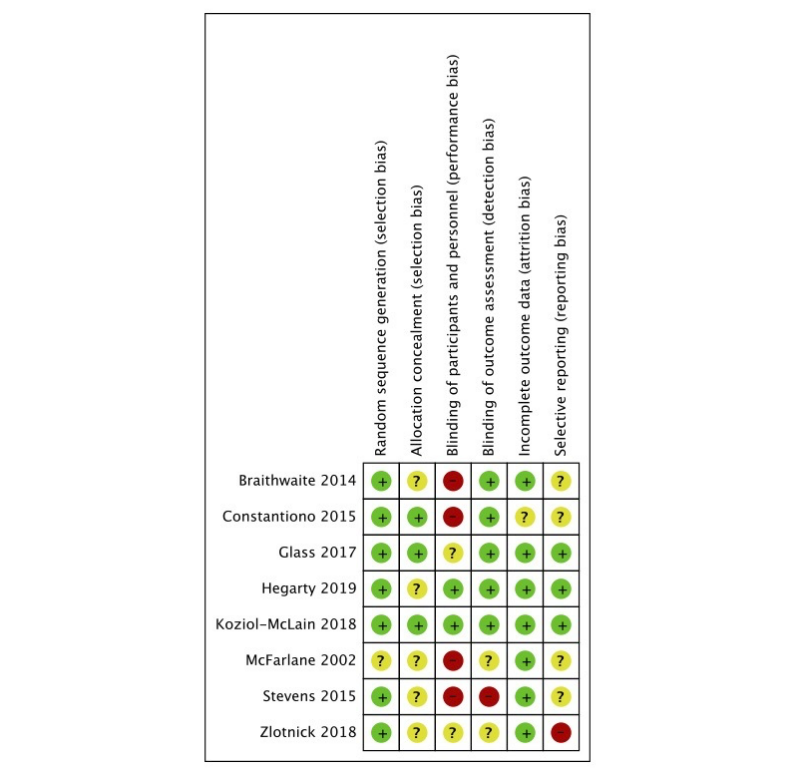
Risk of bias assessment.

Of the 8 published trials, 2 were assessed as overall in low risk of bias [24,25]. In 5 trials, allocation concealment was judged to be unclear, and lack of blinding or unclear description of blinding of personnel resulted in only 2 of the 8 trials being judged as in low risk of performance bias. Further, 4 trials did not have a record in a trial registry, which led to a judgment of unclear risk of reporting bias, and 1 trial had outcomes in the registry not reported in the trial publication, which led to a judgment of high risk of reporting bias (Figure 3, Multimedia Appendix 3).

### Meta-Analysis

**Figure 4:**
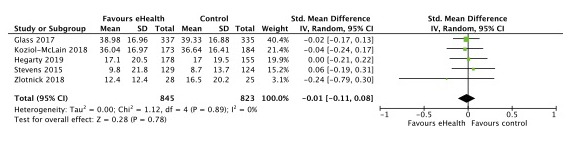
Effect of eHealth versus no eHealth on overall intimate partner violence.

**Figure 5:**
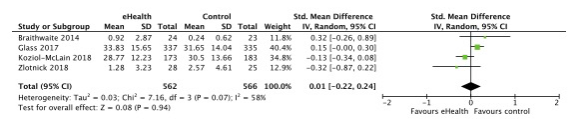
Effect of eHealth versus no eHealth on physical violence.

**Figure 6:**
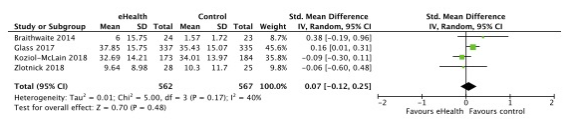
Effect of eHealth versus no eHealth on psychological violence.

**Figure 7:**
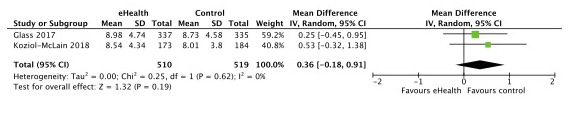
Effect of eHealth versus no eHealth on sexual violence.

Seven published trials were eligible for meta-analysis as they had outcomes on either overall IPV, type-specific IPV, depression, or PTSD [8,24,25,27-29,31]. Five trials (1668 participants) reported data on our primary outcome, overall IPV, and we found no difference in effect of eHealth compared with no eHealth interventions (standardized mean difference [SMD] –0.01; 95% CI –0.11 to 0.08; *I*^2^=0% [Figure 4]). Four trials reported data on physical violence (1128 participants) and psychological violence (1129 participants) [8,25,27,31], and we found no difference in effect of eHealth interventions compared with no eHealth interventions (SMD [physical] 0.01; 95% CI –0.22 to 0.24; *I*^2^=58% [Figure 5]; SMD [psychological] 0.07; 95% CI –0.12 to 0.25; *I*^2^=40% [Figure 6]). Two trials (1029 participants) reported data on sexual violence [8,25], and we found no effect of eHealth interventions compared with no eHealth interventions (mean difference [MD] 0.36; 95% CI –0.18 to 0.91; *I*^2^=0% [Severity of Violence Against Women Scale; Figure 7]).

Five trials reported data on the effect of eHealth on depression (1600 participants) [8,24,25,28,29]. We found no difference in effect of eHealth interventions compared with no eHealth interventions (SMD –0.13; 95% CI –0.37 to 0.11; *I*^2^=78% [Figure S1, Multimedia Appendix 4]). However, as our main analysis showed high statistical heterogeneity, we decided to explore this in a post hoc sensitivity analysis by excluding one small trial with extreme results and a remarkably low standard deviation that also measured depression on a different scale (Patient-Reported Outcomes Measurement Information System) [28] than the other 4 trials, which measured depression on the same scale (Center for Epidemiologic Studies Depression Scale). In our sensitivity analysis (1578 participants), we also found no effect of eHealth interventions compared with no eHealth interventions; however, the heterogeneity disappeared (MD –0.73; 95% CI –2.61 to 1.16; *I*^2^: 0% [Figure S2, Multimedia Appendix 4]). Three trials (1267 participants) reported data on PTSD [8,25,29], and we found no effect of eHealth interventions compared with no eHealth interventions (MD –0.11; 95% CI –1.04 to 0.82; *I*^2^=0% [PTSD Checklist; Figure S3, Multimedia Appendix 4]).

### Subgroup Analyses

We conducted a number of prespecified subgroup analyses on our primary outcome, overall IPV. Our subgroup analysis that compared low risk with high risk of bias trials showed similar results as our primary analysis on overall IPV (SMD_low risk bias_ –0.03; 95% CI –0.15 to 0.10; *I*^2^=0% versus SMD_high risk bias_ 0.01; 95% CI –0.15 to 0.16; *I*^2^=0%; interaction test *P*=.75 [Figure S4, Multimedia Appendix 4]). Similarly, our subgroup analyses showed no effect of eHealth on reduction of IPV, if data was stratified according to type of scale (SMD [Women’s Experience With Battering Scale] –0.03; 95% CI –0.15 to 0.10; *I*^2^=0% vs SMD [Composite Abuse Scale] 0.01; 95% CI –0.15 to 0.16; *I*^2^=0%; interaction test *P*=.75 [Figure S5, Multimedia Appendix 4]), or if data were stratified according to type of eHealth intervention (SMD_telephone support_ 0.06; 95% CI –0.19 to 0.31; *I*^2^=NA vs SMD_online decision aid_ –0.02; 95% CI –0.12 to 0.09; *I*^2^=0% vs SMD_online education_ –0.24; 95% CI –0.79 to 0.30; *I*^2^=NA; interaction test *P*=.59 [Figure S6, Multimedia Appendix 4]). One study of online education had a point estimate of relevant effect size in the favorable direction [27]; however, the confidence interval was wide and the study was judged as high risk of bias.

## Discussion

### Principal Findings

In this systematic review and meta-analysis, we found no evidence that eHealth interventions reduced physical, sexual, or psychological violence, depression, or PTSD compared with no eHealth intervention. We explored if the effect of eHealth interventions varied between type of intervention or the IPV scale used in our subgroup analyses but found no differences. A total of 14 trials set in the United States, Australia, and New Zealand were included in our review. Of the 8 published trials, 2 were assessed as overall in low risk of bias, and 7 trials were eligible to be included in one or more of our meta-analyses. The included studies had considerable heterogeneity in terms of type of eHealth interventions, recruitment strategies, reported outcomes, and outcome measurement tools. While in most analyses this did not result in considerable statistical heterogeneity, this limited our ability to pool the results and identify patterns across studies.

### Comparison With Other Literature

Our findings are somewhat in line with other systematic reviews within this field that partly include eHealth trials. A 2014 Cochrane review that included 13 trials with 3417 participants on interventions to reduce or prevent IPV among pregnant women found that there was lack of consistency in the reported outcomes and therefore meta-analysis was not undertaken [14]. A 2015 Cochrane review that included 13 trials with 2141 participants on advocacy interventions to reduce IPV and promote psychosocial well-being of women also found considerable heterogeneity across trials with a wide range of outcomes (n=25), measurement scales, types of interventions, and time points of outcome measurements. As a result, most of the trials could not be pooled. For the trials they did manage to pool, the authors found no evidence of effect for the majority of violence outcome. None of the studies included in the review were judged to be of good quality, and the authors concluded that it was uncertain how much advocacy interventions benefit women exposed to violence [15].

### Strengths and Limitations

This is the first systematic review focusing on the effect of eHealth interventions on IPV. We conducted a comprehensive literature search that involved both published and unpublished trials. However, our study has some limitations. First, we were unable to include data from unpublished trials and one published trial in our meta-analysis. Nevertheless, our effect estimate was precise for our primary analysis (ie, CI 95% –0.11 to 0.02); hence, a clinically meaningful effect of eHealth interventions on overall IPV appears to be minimal. Second, all the published trials were conducted in high-income countries, which may limit the generalizability. It is plausible that both the attitude toward IPV and the adaptation to eHealth interventions may be affected by local conditions [12]. Third, we chose to limit our meta-analysis to the outcomes overall IPV; physical, sexual, and psychological violence; depression; and PTSD as we saw these outcomes as clinically most relevant. However, our results show that these outcomes were not reported in all trials or necessarily one of the primary outcomes selected by the trial authors. Therefore, it might have been relevant to analyze other proxy measures for IPV (eg, safety behavior or self–efficacy) or other types of violence (eg, financial violence). However, including additional outcomes would increase the risk of a type I error. Fourth, outcome reporting was generally poor, and we found that 4 of the 8 published trials did not have a trial registry record; further, 1 trial did not report all prespecified outcomes. This leaves a concern for selective reporting where outcomes are selected based on the direction of findings. However, such bias generally leads to overestimation of intervention effects and therefore is unlikely to influence our conclusions. Finally, a limited number of trials were eligible to be included in this review. Hence, a future scoping review with broader eligibility criteria may complement this review and provide a more comprehensive understanding of the current state of the literature.

### Implications for Practice and Research

Based on this review, we recommend conducting more high-quality trials within the field of IPV and eHealth to better ascertain the effect of eHealth interventions on IPV and IPV-related outcomes. While we found no effect of eHealth interventions despite their potential to provide a safe space for survivors, it is plausible that eHealth interventions cannot stand alone as an intervention to overcome a complex issue such as IPV. Future research may consider assessing the effect of eHealth in combination with other interventions.

The serious issue of heterogeneity in relation to types of outcomes and outcome measurements in IPV trials suggests that there is currently no consensus on which outcomes are important and how to measure them within in the field. This problem appears to go beyond eHealth and be a general problem within IPV intervention trials [14,15]. Other clinical areas have had similar issues in relation to lack of uniform outcomes, and this has led to initiatives that aim to establish core outcome sets within the fields. The Core Outcomes in Women’s and Newborn Health initiative is an international initiative led by journal editors to harmonize outcome reporting in women’s health research [38,39]. It is part of the Core Outcome Measures in Effectiveness Trials initiative that strives to develop core outcome sets for clinical trials and other types of research [40]. Similarly, the Outcome Measures in Rheumatology initiative has led to the development of core outcome sets in rheumatology [41]. With inspiration from other clinical areas, we therefore recommend establishing an initiative within IPV that strives to develop core outcome sets that as a minimum should be measured and reported within IPV research.

### Conclusions

This systematic review and meta-analysis found no evidence from randomized trials of a beneficial effect of eHealth interventions on overall IPV; physical, sexual, or psychological violence; or depression and PTSD. However, the types of outcomes and how they were measured were very heterogenous across trials, which limited the possibility of pooling results and identifying patterns across studies. More high-quality trials are needed, and we recommend harmonizing outcome reporting in IPV trials by establishing core outcome sets.

## Appendix

Multimedia Appendix 1 Preferred Reporting Items for Systematic Reviews and Meta-Analyses (PRISMA) checklist.

Multimedia Appendix 2 Database search strings.

Multimedia Appendix 3 Risk of bias assessment.

Multimedia Appendix 4 Additional meta-analyses.

## Abbreviations

IPV: intimate partner violence
MD: mean difference
PRISMA: Preferred Reporting Items for Systematic Reviews and Meta-Analyses
PROSPERO: International Prospective Register for Systematic Reviews
PTSD: posttraumatic stress disorder
SMD: standardized mean difference
