# Supplementary material for: Intimate Partner Violence and Electronic Health Interventions: Systematic Review and Meta-Analysis of Randomized Trials
Source: J Med Internet Res. 2020 Dec 11;22(12):e22361. doi: 10.2196/22361 (PMC7762681; doi:10.2196/22361)
Supplement: Multimedia Appendix 2 [file jmir_v22i12e22361_app2.pdf]

Multimedia appendix 2: Database search strings

MEDLINE (Ovid)

Ovid MEDLINE(R) and Epub Ahead of Print, In-Process & Other Non-Indexed Citations and Daily 1946 to March 29, 2019

Search date: 01.04.2019

# ID	Search	Result
1	Exp Intimate Partner Violence/	8632
2	Exp Domestic Violence/	42088
3	Exp Spouse Abuse/	7231
4	Exp Battered Women/	2572
5	(((partner* or woman or women or female* or wife or wives or mother* or spouse* or spousal or pregnant or marital or domestic or couple* or marriage or family or families or dating*) adj3 (violen* or batter* or abuse* or aggression* or coercion* or assault* or exploitation* or slap* or kick* or hit* or beat* or insult* or harass* or stalk* or threat* or intimidat* or humiliat* or rape or homicide*)) or ((adolescent* or teen*or youth*) adj3 (female* or girl* or relationship* or couple* or dating*) adj3 (violen* or batter* or abuse* or aggression* or coercion* or assault* or exploitation* or slap* or kick* or hit* or beat* or insult* or harass* or stalk* or threat* or intimidat* or humiliat* or rape or homicide*)) or ((physical* or sexual* or psychological* or emotional* or intimate) adj3 (violence* or batter* abuse* or aggression* or coercion* or assault* or exploitation*) adj3 (partner*or wife or wives or woman or women or mother* or pregnant or female* or adolescent* or teen*or youth* or spouse* or spousal or marital or domestic or couple* or marriage or family or families or dating*)) or intimate-partner violence or intimate partner violence or domestic violence).mp. [mp=title, abstract, original title, name of substance word, subject heading word, floating sub-heading word, keyword heading word, organism supplementary concept word, protocol supplementary concept word, rare disease supplementary concept word, unique identifier, synonyms]	32597
6	1 or 2 or 3 or 4 or 5	59675
7	Exp Telemedicine/	24578
8	Exp Telecommunications/	84144
9	Exp Bloggin/	908
10	Exp Mass Media/	44170
11	Exp Social Media/	5620
12	Exp Access to Information/	7021
13	Exp Reminder Systems/	3187
14	Exp Social Networking/	2568
15	Exp Computers/	75527
16	Exp Electronic Mail/	2512
17	Exp Internet/	720084
18	Exp internet Access/	16
19	Exp Web Browser/	904
20	Exp Online Social Networking/	23
21	Exp Telephone/	20511
22	Exp Cell Phone/	9312
23	Exp Smartphone/	2764
24	Exp Mobile Applications/	3909
25	Exp Text Messaging/	2194
26	Exp Video-Audio Media/	20466
27	Exp Webcasts/	683
28	Exp Webcasts as Topic/	303
29	Exp Television/	31140
30	Exp Radio/	2153
31	Exp Tape Recording/	15332
32	Exp Videoconferencing/	1622

33	Exp Wireless Technology/	3033
34	Exp Video Recording/	38538
35	Tablets/	21444
36	Exp Hotlines/	2611
37	(((cell* or mobile or smart) adj3 (phone* or telephone*)) or ((phone* or telephone* or smartphone* or cell* or mobile) adj3 (call* or text* or messag* or SMS or reminder*)) or ((internet or online or on-line or web or website* or webcast* or wireless or electronic*) adj3 (technology or technologies or device* or information* or application* or podcast*)) or ((video* or tape* or audio*) adj3 (conference* or recording*)) or ((Internet or web* or online or on-line or mail*) adj3 (survey* or information* or access*)) or ((social* or telecommunication* or communication* or mass) adj3 (media or medium or network*)) or telemedicine or eHealth or e-health or tele-health or telenursing or mHealth or mobile health or broadcast or twitter or facebook or facetime or whatsapp or skype or smartphone* or computer* or PC* or iPad* or tablet* or mobile*or mail* or e-mail* or email* or blogging* or radio or television or hotline).mp. [mp=title, abstract, original title, name of substance word, subject heading word, floating sub-heading word, keyword heading word, organism supplementary concept word, protocol supplementary concept word, rare disease supplementary concept word, unique identifier, synonyms]	1863119
38	7 or 8 or 9 or 10 or 11 or 12 or 13 or 14 or 15 or 16 or 17 or 18 or 19 or 20 or 21 or 22 or 23 or 24 or 25 or 26 or 27 or 28 or 29 or 30 or 31 or 32 or 33 or 34 or 35 or 36 or 37	1960135
39	(((singl* or doubl* or treb* or tripl*) adj (blind*3 or mask*3)).tw or (allocated adj2 random).tw. or (clin* adj25 trial*).ti,ab. or (clinic* adj trial*1).tw. or (double-blind* or random*).af. or clinical trial.pt. or clinical trials as topic.sh. or controlled clinical trial.pt. or double blind method.sh. or single blind method.sh. or double-blind method.sh. or single-blind method.sh. or drug therapy.fs. or exp clinical trials as topic/ or exp research design/ or placebo*.tw. or placebos.sh. or practice guideline.pt. or random allocation.sh. or random*.tw. or random.af. or randomized controlled trial.pt. or randomized controlled trials as topic.sh. or randomized.ab. or randomly allocated.tw. or randomly.ab. or single-blind method.sh. or trial.ab. or trial.ti.) not (case report.tw. or letter.pt. or historical article.pt. or review of reported cases.pt or multicase review.pt.)	3515863
40	6 and 39 and 40	448

EMBASE (Ovid)

Embase Classic + Embase 1947 to 2019 March 29

Search date: 01.04.2019

# ID	Search	Result
1	Exp partner violence/	10794
2	Exp domestic violence/	56391
3	Exp dating violence/	360
4	Exp battered woman/	3199
5	Exp marital rape/	42
6	Exp family violence/	3738
7	(((partner* or woman or women or female* or wife or wives or mother* or spouse* or spousal or pregnant or marital or domestic or couple* or marriage or family or families or dating*) adj3 (violen* or batter* or abuse* or aggression* or coercion* or assault* or exploitation* or slap* or kick* or hit* or beat* or insult* or harass* or stalk* or threat* or intimidat* or humiliat* or rape or homicide*)) or ((adolescent* or teen*or youth*) adj3 (female* or girl* or relationship* or couple* or dating*) adj3 (violen* or batter* or abuse* or aggression* or coercion* or assault* or exploitation* or slap* or kick* or hit* or beat* or insult* or harass* or stalk* or threat* or intimidat* or humiliat* or rape or homicide*)) or ((physical* or sexual* or psychological* or emotional* or intimate) adj3 (violence* or batter* abuse* or aggression* or coercion* or assault* or exploitation*) adj3 (partner*or wife or wives or woman or women or mother* or pregnant or female* or adolescent* or teen*or youth* or spouse* or spousal or marital or domestic or couple* or marriage or family or families or dating*)) or intimate-partner violence or intimate partner violence or domestic violence).mp. [mp=title, abstract,	40546

	heading word, drug trade name, original title, device manufacturer, drug manufacturer, device trade name, keyword, floating subheading word, candidate term word]	
8	1 or 2 or 3 or 4 or 5 or 6 or 7	72557
9	Exp telehealth/	38554
10	Exp telemedicine/	34463
11	Exp mobile phone/	22422
12	Exp smartphone/	7810
13	Exp telephone/	35852
14	Exp text messaging/	3960
15	Exp mobile application/	8054
16	Exp mobile health application/	346
17	Exp telecommunication/	62802
18	Exp teleconference/	1308
19	Exp voice mail/	79
20	Exp blogging/	269
21	Exp hotline/	297
22	Exp e-mail/	19244
23	Exp internet/	101973
24	Exp mass medium/	17869
25	Exp radio/	354
26	Exp television/	16851
27	Exp social media/	14522
28	Exp videoconferencing/	3229
29	Exp webcast/	316
30	Exp wireless communication/	4519
31	Exp computer network/	14547
32	Exp computer/	147549
33	Exp access to information/	18715
34	Exp videorecording/	75376
35	Exp reminder system/	2390
36	Exp social network/	13799
37	Exp online system/	24111
38	Exp tablet machine/	300
39	Exp tablet/	45815
40	Exp computer system/	25415
41	(((cell* or mobile or smart) adj3 (phone* or telephone*)) or ((phone* or telephone* or smartphone* or cell* or mobile) adj3 (call* or text* or messag* or SMS or reminder*)) or ((internet or online or on-line or web or website* or webcast* or wireless or electronic*) adj3 (technology or technologies or device* or information* or application* or podcast*)) or ((video* or tape* or audio*) adj3 (conference* or recording*)) or ((Internet or web* or online or on-line or mail*) adj3 (survey* or information* or access*)) or ((social* or telecommunication* or communication* or mass) adj3 (media or medium or network*)) or telemedicine or eHealth or e-health or tele-health or telenursing or mHealth or mobile health or broadcast or twitter or facebook or facetime or whatsapp or skype or smartphone* or computer* or PC* or iPad* or tablet* or mobile*or mail* or e-mail* or email* or blogging* or radio or television or hotline).mp. [mp=title, abstract, heading word, drug trade name, original title, device manufacturer, drug manufacturer, device trade name, keyword, floating subheading word, candidate term word]	4078190
42	9 or 10 or 11 or 12 or 13 or 14 or 15 or 16 or 17 or 18 or 19 or 20 or 21 or 22 or 23 or 24 or 25 or 26 or 27 or 28 or 29 or 30 or 31 or 32 or 33 or 34 or 35 or 36 or 37 or 38 or 39 or 40 or 41	4243342
43	(((singl* or doubl* or treb* or tripl*) adj (blind*3 or mask$3)) or (allocated adj2 random)).tw. or (clin* adj25 trial*).ti,ab. or (clinic: adj trial$1).tw. or (double-blind* or random*).af. or exp "clinical trial (topic)"/ or exp double blind procedure/ or exp single blind procedure/ or exp triple blind procedure/ or placebo*.tw. or exp placebo/ or exp randomization/ or Random.af. or Random*.tw. or exp "randomized controlled trial (topic)"/ or randomized.ab. or randomly allocated.tw. or randomly.ab. or trial.ab. or trial.ti. or exp "controlled clinical trial (topic)"/ or randomized controlled trial/ or "randomized controlled trial (topic)"/ or exp "controlled clinical trial"/	2546061
44	8 and 42 and 43	1004

PsycInfo (Ovid)

PsycINFO 1806 to March Week 4 2019

Search date: 01.04.2019

ID	Search	Result
1	Exp Intimate Partner Violence/	10763
2	Exp Domestic Violence/	10936
3	Exp Partner Abuse/	10763
4	Exp Battered Females/	3072
5	(((partner* or woman or women or female* or wife or wives or mother* or spouse* or spousal or pregnant or marital or domestic or couple* or marriage or family or families or dating*) adj3 (violen* or batter* or abuse* or aggression* or coercion* or assault* or exploitation* or slap* or kick* or hit* or beat* or insult* or harass* or stalk* or threat* or intimidat* or humiliat* or rape or homicide*)) or ((adolescent* or teen*or youth*) adj3 (female* or girl* or relationship* or couple* or dating*) adj3 (violen* or batter* or abuse* or aggression* or coercion* or assault* or exploitation* or slap* or kick* or hit* or beat* or insult* or harass* or stalk* or threat* or intimidat* or humiliat* or rape or homicide*)) or ((physical* or sexual* or psychological* or emotional* or intimate) adj3 (violence* or batter* abuse* or aggression* or coercion* or assault* or exploitation*) adj3 (partner*or wife or wives or woman or women or mother* or pregnant or female* or adolescent* or teen*or youth* or spouse* or spousal or marital or domestic or couple* or marriage or family or families or dating*)) or intimate-partner violence or intimate partner violence or domestic violence).mp. [mp=title, abstract, original title, name of substance word, subject heading word, floating sub-heading word, keyword heading word, organism supplementary concept word, protocol supplementary concept word, rare disease supplementary concept word, unique identifier, synonyms]	47417
6	1 or 2 or 3 or 4 or 5	47417
7	Exp Telemedicine/	4687
8	Exp Mobile Devices/	2180
9	Exp Cellular Phones/	0
10	Exp Telephone Systems/	1945
11	Exp Text Messaging/	739
12	Exp MESSAGES/	6797
13	Exp TELEPHONE SURVEYS/	506
14	Exp Telecommunications Media/	12433
15	Exp TELECONFERENCING/	862
16	Exp RADIO/	1094
17	Exp TELEVISION/	8349
18	Exp INTERNET/	28297
19	Exp Internet Usage/	10604
20	Exp Online Social Networks/	6931
21	Exp Computer Mediated Communication/	6756
22	Exp Teleconferencing/	862
23	Exp Social Media/	11862
24	Exp BLOG/	419
25	Exp Electronic Communication/	21021
26	Exp MAIL SURVEYS/	303
27	Exp COMPUTERS/	18912
28	Exp Computer Applications/	63229
29	Exp WEBSITES/	5007
30	Exp GROUPWARE/	227
31	Exp Hot Line Services/	1002
32	Exp Computer Software/	14060
33	Exp DIGITAL VIDEO/	1665
34	(((cell* or mobile or smart) adj3 (phone* or telephone*)) or ((phone* or telephone* or smartphone* or cell* or mobile) adj3 (call* or text* or messag* or SMS or reminder*)) or ((internet or online or on-line or web or website* or webcast* or wireless or electronic*) adj3 (technology or technologies or device* or information* or application* or podcast*)) or	2955667

	((video* or tape* or audio*) adj3 (conference* or recording*)) or ((Internet or web* or online or on-line or mail*) adj3 (survey* or information* or access*)) or ((social* or telecommunication* or communication* or mass) adj3 (media or medium or network*)) or telemedicine or eHealth or e-health or tele-health or telenursing or mHealth or mobile health or broadcast or twitter or facebook or facetime or whatsapp or skype or smartphone* or computer* or PC* or iPad* or tablet* or mobile*or mail* or e-mail* or email* or blogging* or radio or television or hotline).mp. [mp=title, abstract, original title, name of substance word, subject heading word, floating sub-heading word, keyword heading word, organism supplementary concept word, protocol supplementary concept word, rare disease supplementary concept word, unique identifier, synonyms]	
35	7 or 8 or 9 or 10 or 11 or 12 or 13 or 14 or 15 or 16 or 17 or 18 or 19 or 20 or 21 or 22 or 23 or 24 or 25 or 26 or 27 or 28 or 29 or 30 or 31 or 32 or 33 or 34	338352
36	Exp Treatment Effectiveness Evaluation/	23193
37	Exp Treatment Outcomes/	36495
38	Exp PLACEBO/	5216
39	Exp Followup Studies/	12366
40	(placebo* or random* or comparative stud* or (clinical adj4 trial*) or (research adj4 design) or (evaluat* adj4 stud*) or (prospectiv* adj4 stud*) or ((singl* or doubl* or trebl* or tripl*) adj4 (blind* or mask))).mp. [mp=title, abstract, heading word, table of contents, key concepts, original title, tests & measures]	354330
41	37 or 38 or 39 or 40 or 41	403107
42	6 and 36 and 42	276

Cochrane Library (CENTRAL)

Search date: 01.04.2019

ID	Search	Result
#1	MeSH descriptor: [Domestic Violence] explode all trees	813
#2	MeSH descriptor: [Spouse Abuse] explode all trees	199
#3	MeSH descriptor: [Sexual Partners] explode all trees	575
#4	MeSH descriptor: [Battered Women] explode all trees	63
#5	(((partner* or woman or women or female* or wife or wives or mother* or spouse* or spousal or pregnant or marital or domestic or couple* or marriage or family or families or dating*) NEAR/2 (violen* or batter* or abuse* or aggression* or coercion* or assault* or exploitation* or slap* or kick* or hit* or beat* or insult* or harass* or stalk* or threat* or intimidat* or humiliat* or rape or homicide*)) or ((adolescent* or teen*or youth*) NEAR/2 (female* or girl* or relationship* or couple* or dating*) NEAR/2 (violen* or batter* or abuse* or aggression* or coercion* or assault* or exploitation* or slap* or kick* or hit* or beat* or insult* or harass* or stalk* or threat* or intimidat* or humiliat* or rape or homicide*)) or ((physical* or sexual* or psychological* or emotional* or intimate) NEAR/2 (violence* or batter* abuse* or aggression* or coercion* or assault* or exploitation*) NEAR/2 (partner*or wife or wives or woman or women or mother* or pregnant or female* or adolescent* or teen*or youth* or spouse* or spousal or marital or domestic or couple* or marriage or family or families or dating*)) or intimate-partner violence or intimate partner violence or domestic violence):ti,ab,kw	1925
#6	#1 or #2 or #3 or #4 or #5	2824
#7	MeSH descriptor: [Telemedicine] explode all trees	2044
#8	MeSH descriptor: [Cell Phone] explode all trees	1083
#9	MeSH descriptor: [Telephone] explode all trees	2961
#10	MeSH descriptor: [Smartphone] explode all trees	219
#11	MeSH descriptor: [Reminder System] explode all trees	836
#12	MeSH descriptor: [Text Messaging] explode all trees	630
#13	MeSH descriptor: [Hotlines] explode all trees	150
#14	MeSH descriptor: [Social Media] explode all trees	99
#15	MeSH descriptor: [Blogging] explode all trees	13
#16	MeSH descriptor: [Mass Media] explode all trees	1781
#17	MeSH descriptor: [Telecommunications] explode all trees	5485

#18	MeSH descriptor: [Online Social Networking] explode all trees	1
#19	MeSH descriptor: [Electronic Mail] explode all trees	299
#20	MeSH descriptor: [Computers, Mainframe] explode all trees	0
#21	MeSH descriptor: [Internet Access] explode all trees	1
#22	MeSH descriptor: [Radio] explode all trees	28
#23	MeSH descriptor: [Television] explode all trees	1500
#24	MeSH descriptor: [Tape Recording] explode all trees	1178
#25	MeSH descriptor: [Video-Audio Media] explode all trees	0
#26	MeSH descriptor: [Webcasts as Topic] explode all trees	20
#27	(((cell* or mobile or smart) NEAR/2 (phone* or telephone*)) or ((phone* or telephone* or smartphone* or cell* or mobile) NEAR/2 (call* or text* or messag* or SMS or reminder*)) or ((internet or online or on-line or web or website* or webcast* or wireless or electronic*) NEAR/2 (technology or technologies or device* or information* or application* or podcast*)) or ((video* or tape* or audio*) NEAR/2 (conference* or recording*)) or ((Internet or web* or online or on-line or mail*) NEAR/2 (survey* or information* or access*)) or ((social* or telecommunication* or communication* or mass) NEAR/2 (media or medium or network*)) or telemedicine or eHealth or e-health or tele-health or telenursing or mHealth or mobile health or broadcast or twitter or facebook or facetime or whatsapp or skype or smartphone* or computer* or PC* or iPad* or tablet* or mobile*or mail* or e-mail* or email* or blogging* or radio or television or hotline):ti,ab,kw	160855
#28	#7 or #8 or #9 or #10 or #11 or #12 or #13 or #14 or #15 or #16 or #17 or #18 or #19 or #20 or #21 or #22 or #23 or #24 or #25 or #26 or #27	162812
#29	#6 and #28	448

Scopus (Elsevier)

Search date: 01.04.2019

ID	Search	Result
1	(TITLE-ABS-KEY(((partner* or woman or women or female* or wife or wives or mother* or spouse* or spousal or marital or domestic or couple* or marriage or family or families or dating*) W/2 (violen* or batter* or abuse* or aggression* or coercion* or assault* or rape)))) OR (TITLE-ABS-KEY(((adolescent* or teen* or youth*) W/2 (female* or girl* or relationship* or couple* or dating*) W/2 (violen* or batter* or abuse* or aggression* or coercion* or assault* or rape)))) OR (TITLE-ABS-KEY(((partner* OR woman OR women OR female* OR wife OR wives OR mother* OR spouse* OR spousal) W/2 (violence* OR batter* OR abuse* OR aggression* OR coercion* OR assault* OR exploitation*) W/2 (physical* OR sexual* OR psychological* OR emotional* OR intimate)))) OR (TITLE-ABS-KEY(intimate-partner violence or intimate partner violence OR domestic violence))	56,405
2	(TITLE-ABS-KEY(((cell* or mobile or smart) W/2 (phone* or telephone*))) OR TITLE-ABS-KEY(((phone* or telephone* or smartphone* or cell* or mobile) W/2 (call* or text* or messag* or SMS or reminder*))) OR TITLE-ABS-KEY(((internet or online or on-line or web or website* or webcast* or wireless or electronic*) W/2 (technology or technologies or device* or information* or application* or podcast*))) OR TITLE-ABS-KEY(((video* or tape* or audio*) W/2 (conference* or recording*))) OR TITLE-ABS-KEY(((Internet or web* or online or on-line or mail*) W/2 (survey* or information* or access*))) OR TITLE-ABS-KEY(((social* or telecommunication* or communication* or mass) W/2 (media or medium or network*))) OR TITLE-ABS-KEY(telemedicine or eHealth or e-health or tele-health or telenursing or mHealth or mobile health or broadcast or twitter or facebook or facetime or whatsapp or skype or smartphone* or computer* or PC* or iPad* or tablet* or mobile*or mail* or e-mail* or email))	974,227
3	(INDEXTERMS ("clinical trials" OR "clinical trials as a topic" OR "randomized controlled trial" OR "Randomized Controlled Trials as Topic" OR "controlled clinical trial" OR "Controlled Clinical Trials" OR "random allocation" OR "Double-Blind Method" OR "Single-Blind Method" OR "Cross-Over Studies" OR "Placebos" OR "multicenter study" OR "double blind procedure" OR "single blind procedure" OR "crossover procedure" OR "clinical trial" OR "controlled study" OR "randomization" OR "placebo")) OR (TITLE-ABS-KEY ((	7,050,115

	"clinical trials" OR "clinical trials as a topic" OR "randomized controlled trial" OR "Randomized Controlled Trials as Topic" OR "controlled clinical trial" OR "Controlled Clinical Trials as Topic" OR "random allocation" OR "randomly allocated" OR "allocated randomly" OR "Double-Blind Method" OR "Single-Blind Method" OR "Cross-Over Studies" OR "Placebos" OR "cross-over trial" OR "single blind" OR "double blind" OR "factorial design" OR "factorial trial"))) OR (TITLE-ABS (clinical trial* OR trial* OR rct* OR random* OR blind*))	
4	#1 AND #2 AND #3	214

Global Health Library

Search date: 01.04.2019

ID	Search	Result
S1	DE “spouse abuse”	1,286
S2	((partner* or woman or women or female* or wife or wives or mother* or spouse* or spousal or pregnant or marital or domestic or couple* or marriage or family or families or dating*) N/2 (violen* or batter* or abuse* or aggression* or coercion* or assault* or exploitation* or slap* or kick* or hit* or beat* or insult* or harass* or stalk* or threat* or intimidat* or humiliat* or rape or homicide*)) or ((adolescent* or teen*or youth*) N/2 (female* or girl* or relationship* or couple* or dating*) N/2 (violen* or batter* or abuse* or aggression* or coercion* or assault* or exploitation* or slap* or kick* or hit* or beat* or insult* or harass* or stalk* or threat* or intimidat* or humiliat* or rape or homicide*)) or ((physical* or sexual* or psychological* or emotional* or intimate) N/2 (violence* or batter* abuse* or aggression* or coercion* or assault* or exploitation*) N/2 (partner*or wife or wives or woman or women or mother* or pregnant or female* or adolescent* or teen*or youth* or spouse* or spousal or marital or domestic or couple* or marriage or family or families or dating*)) or intimate-partner violence or intimate partner violence or domestic violence	3,383
S3	S1 OR S1	3,628
S4	DE “telecommunications” OR DE “internet”	8,375
S5	DE “telephones”	1,202
S6	DE “television”	2,626
S7	DE “radio” OR DE “educational radio”	364
S8	DE “internet”	7,492
S9	DE “mass media”	3,801
S10	DE “computer software”	3,946
S11	DE “video recordings” OR DE “videodiscs” OR DE “videotapes”	454
S12	((cell* or mobile or smart) N/2 (phone* or telephone*)) or ((phone* or telephone* or smartphone* or cell* or mobile) N/2 (call* or text* or messag* or SMS or reminder*)) or ((internet or online or on-line or web or website* or webcast* or wireless or electronic*) N/2 (technology or technologies or device* or information* or application* or podcast*)) or ((video* or tape* or audio*) N/2 (conference* or recording*)) or ((Internet or web* or online or on-line or mail*) N/2 (survey* or information* or access*)) or ((social* or telecommunication* or communication* or mass) N/2 (media or medium or network*)) or telemedicine or eHealth or e-health or tele-health or telenursing or mHealth or mobile health or broadcast or twitter or facebook or facetime or whatsapp or skype or smartphone* or computer* or PC* or iPad* or tablet* or mobile*or mail* or e-mail* or email* or blogging* or radio or television or hotline	219,596
S13	S4 OR S5 OR S6 OR S7 OR S8 OR S9 OR S10 OR S11 OR S12	228,493
S14	DE “reviews”	161,488
S15	DE “placebos”	1,254
S16	DE “clinical trials” OR DE “randomized controlled trials”	49,635
S17	AB "random allocation" OR AB "random allocations" OR AB "random allocated" OR AB "randomly allocated" OR AB "clinical trials" OR AB placebo OR AB placebos OR AB "drug therapy" OR "review of reported cases" OR "multicase review" OR "practice guideline"	58,522
S18	"randomized controlled trial" OR "Clinical Trial" OR "Controlled Clinical Trial" OR AB "single blind" OR AB "single blinded" OR AB "single masked" OR AB "double blind" OR AB "double blinded" OR AB "double masked" OR AB "triple blind" OR "triple blinded" OR	45,887

	"triple masked"	
S19	S14 OR S15 OR S16 OR S17 OR S18	257,081
S20	(S14 OR S15 OR S16 OR S17 OR S18) AND (S3 AND S13 AND S19)	16

ClinicalTrials.gov

Search date: 01.04.2019

Search	Result
intimate partner violence OR partner* violence OR partner* homicide OR woman violence OR women violence OR women abuse OR woman abuse OR female* violence OR female* abuse OR battered women OR wife violence OR wives violence OR wife abuse OR wives abuse OR mother* violence OR mother* abuse OR spouse* violence OR spouse* abuse OR marital violence OR marital abuse OR domestic violence OR domestic abuse OR family violence OR families violence OR family abuse OR families abuse OR dating* violence OR marital rape OR wife beating OR relationship violence OR relationship abuse OR teen* violence OR youth* violence OR adolescent* violence OR teen* abuse OR youth* abuse adolescent *abuse	2,673
telemedicine or eHealth or e-health or tele-health or telenursing or mHealth or mobile health OR cell* phone OR mobile* phone OR mobile* telephone OR smart phone OR smartphone* OR phone call* OR telephone call* OR smartphone* call OR mobile call* OR text* message* OR phone* messag* OR phone* text* OR SMS or SMS reminder* OR short message service OR internet technology or internet technologies OR internet information* OR internet application* OR online technology OR online technologies OR on-line technology OR online information* OR on-line information* OR online application* OR on-line application* OR web technology OR web technologies OR web information* OR webcast* technology OR webcast technologies OR wireless technology OR wireless technologies OR podcast OR electronic* device OR electronic* information OR video* conference OR videocoferenc* OR tape* recording OR audio* recording OR internet survey OR web* survey OR mail* survey OR internet access or online access or on-line access OR social* media OR social* medium OR social* network* OR telecommunication* media OR telecommunication* medium OR communication* media OR communication* medium OR broadcast OR twitter OR facebook OR facetime OR whatsapp OR skype OR smartphone* OR computer* OR PC* OR iPad* OR tablet* OR mobile* OR mail* OR e-mail* OR email* OR blogging* OR radio OR television OR hotline	9,228
(intimate partner violence OR partner* violence OR partner* homicide OR woman violence OR women violence OR women abuse OR woman abuse OR female* violence OR female* abuse OR battered women OR wife violence OR wives violence OR wife abuse OR wives abuse OR mother* violence OR mother* abuse OR spouse* violence OR spouse* abuse OR marital violence OR marital abuse OR domestic violence OR domestic abuse OR family violence OR families violence OR family abuse OR families abuse OR dating* violence OR marital rape OR wife beating OR relationship violence OR relationship abuse OR teen* violence OR youth* violence OR adolescent* violence OR teen* abuse OR youth* abuse adolescent *abuse) AND (telemedicine or eHealth or e-health or tele-health or telenursing or mHealth or mobile health OR cell* phone OR mobile* phone OR mobile* telephone OR smart phone OR smartphone* OR phone call* OR telephone call* OR smartphone* call OR mobile call* OR text* message* OR phone* messag* OR phone* text* OR SMS or SMS reminder* OR short message service OR internet technology or internet technologies OR internet information* OR internet application* OR online technology OR online technologies OR on-line technology OR online information* OR on-line information* OR online application* OR on-line application* OR web technology OR web technologies OR web information* OR webcast* technology OR webcast technologies OR wireless technology OR wireless technologies OR podcast OR electronic* device OR electronic* information OR video* conference OR videocoferenc* OR tape* recording OR audio* recording OR internet survey OR web* survey OR mail* survey OR internet access or online access or on-line access OR social* media OR social* medium OR social* network* OR telecommunication* media OR telecommunication* medium OR communication* media OR communication* medium OR broadcast OR twitter OR facebook OR facetime OR whatsapp OR skype OR smartphone* OR computer* OR PC* OR iPad* OR tablet* OR mobile* OR mail* OR e-mail* OR email* OR blogging* OR radio OR television OR hotline)	433

International Clinical Trial Registry Platform (ICTRP)

Search date: 01.04.2019

Search	Result
(Partner violence or partner abuse or domestic violence or spouse abuse or spousal abuse or marital violence or family violence or battered women) AND (Telemedicine or ehealth or technology or mHealth or mobile health or phone or mobile or cell or text or video or message or SMS or computer or online or survey or on-line or email* or phone application or web or podcast)	1
