# Supplementary material for: Intimate Partner Violence and Electronic Health Interventions: Systematic Review and Meta-Analysis of Randomized Trials
Source: J Med Internet Res. 2020 Dec 11;22(12):e22361. doi: 10.2196/22361 (PMC7762681; doi:10.2196/22361)
Supplement: Multimedia Appendix 3 [file jmir_v22i12e22361_app3.pdf]

Multimedia Appendix 3: Risk of bias assessment

Braithwaite, 2014

Bias	Authors' judgement	Support for judgement
Random sequence generation (selection bias)	Low risk	"Couples were randomly assigned to condition using a computer-generated randomization list."
Allocation concealment (selection bias)	Unclear risk	Not stated.
Blinding of participants and personnel (performance bias)	High risk	Married coulples who showed interest to participate in the study were explained that the study was designed to "understand the course of marriage", and that it would include "takinng part in a computer-based presentation that educates you about the relationship"<br><br>"Participants were blind to condition, but the experimenter was not. "
Blinding of outcome assessment (detection bias)	Low risk	"Participant completed the Revised Conflict Tactics Scale (CTS-2) at baseline and at each of the follow-up assessment. […] The CTS-2 provides self and partner-reported account of IPV […]. We used the CTS-2 total score as our outcome (coded as directed in Straus et al, 1996), which includes items for both minor and severe IPV."
Incomplete outcome data (attrition bias)	Low risk	All participants were included in the analyses regardless of whether or not they completed the six weeks of the intervention and or follow-up assessments. Data was analyzed as intention-to-treat. 25 couples were assigned to the control group, and 24 completed first follow-up and 23 the second follow-up. 26 couples were assigned to intervention, and 25 completed first follow-up and 24 the second followup.
Selective reporting (reporting bias)	Unclear risk	No Protocol or registry information available (author has not responded to request for protocol).

Constantino, 2015

Bias	Authors' judgement	Support for judgement
Random sequence generation (selection bias)	Low risk	"Participants were randomly assigned to one of three groups by permuted block randomization."
Allocation concealment (selection bias)	Low risk	"The block randomization table was kept at a centralized location in a locked filing cabinet.[…] The designation of numbers for intervention conditions (i.e., 1= ONL, 2=FTF, 3=WLC), were concealed from data collectors and the statistician."
Blinding of participants and personnel (performance bias)	High risk	Participants were not blinded due to type of intervention. It is not stated if personnel is blinded, however, it is described that the the PI saw the participants in the the waitlist control group twice, and that the the PI sent a triggered email to participants in the the online group, and that a nurse interventionist led the face-to-face session. Hence, it appears personnel was not blinded.
Blinding of outcome assessment (detection bias)	Low risk	Participants completed a researcher-developed IPV experience questionnaire for IPV; the PROMIS 1.0 short for anxiety, depression and anger; and the personale

		resource questionnaire to measure availability of personal support.
Incomplete outcome data (attrition bias)	Unclear risk	The number listed in the article are not all clear. 38 participants were recruited, 32 enrolled for randomization. Reasons for exclusions are stated. Number for each participant vs sociodemographic characteristics does not fit the number of women included, example: WLC=11 (White:2, Black:3, Asian:5). Also, randomization to three groups (n=32) but post-assessment (n=33). Pre- and post-assessment well described for all outcomes.<br><br>For the wailist control group it is stated, that: "Only four of the 11 participants in the WLC group chose to receive the HELPP invention online; however, only two participants received the first three modules (i.e. first 3 weeks), and neither completed all six modules.".
Selective reporting (reporting bias)	Unclear risk	No Protocol or registry information available (author has not responded to request for protocol).

Glass, 2017

Bias	Authors' judgement	Support for judgement
Random sequence generation (selection bias)	Low risk	"Computerized blocked randomization provided intrastate stratification and for participants with children (aged <18 years) at home, ensuring each state's groups remained relatively balanced."<br><br>"Four academic centers conducted this community-based RCT with a one-to-one allocation ratio."
Allocation concealment (selection bias)	Low risk	"The randomization sequence (concealed from research assistants (RAs) was programmed into a secure tracking database separate from the study website by the study programmer, who had no participant contact.
Blinding of participants and personnel (performance bias)	Unclear	Participant were blinded to group assignment. It is not stated if personnel is blinded.
Blinding of outcome assessment (detection bias)	Low risk	All participants completed measures via the secure website at baseline, 6 months and 12 months. All were self-reported.
Incomplete outcome data (attrition bias)/	Low risk	Data was analyzed as intention-to-treat. "Missing data for intervention and control groups (accounting for attrition and incomplete responses) was 9.0% and 5.3% (6 months) and 8.5% and 9.2% (12 months), respectively."
Selective reporting (reporting bias)	Low risk	Trial registered prospectively on ClinGov: NCT01312103 and the protocol of a replical trial conducted in New Zealand is published in BMC Public Health. (Koziol-Mclane, 2018) Outcome measured stated on Clingov:<br>Primary outcomes<br>• Severity of violence against women (6m) – scale not specified but contains 46 items<br>• Women's experience with battering (6m) – WEB scale<br>• Safety behavior checklist (6m)<br>• Decisional conflict scale (6m)<br>Secondary outcomes<br>• Depression – CESDS-R scale (6m)

		• PTSD checklist<br>In the article decisional conflict (DCS), safety behavour, safety planning, IPV exposure (SVAWS + WEB), depression (CEDS-R), and PTSD was measured.There is another article linked to the clingov ID (Eden, 2015) where the following outcomes are addressed: deciosional conflict (measured on DSC), danger assessment (measured on DA scale); priority weights, and relationship intentions.

Hegarty, 2019

Bias	Authors' judgement	Support for judgement
Random sequence generation (selection bias)	Low risk	"Participants were randomly assigned 1:1 by computer to receive either control or intervention website. An automated computerized algorithm for simple 1:1 randomisation as used, with no stratification".
Allocation concealment (selection bias)	Unclear	"As the initial portion of the website containing the baseline questions was identifcal for both groups, there was no way for women to tell which group they had been allocated to. Women were masked to treatment allocation, although it is possible that some may have guessed which website they were receiving.
Blinding of participants and personnel (performance bias)	Low risk	"All the research team were masked to participant allocation until after analysis of the 12-month data."
Blinding of outcome assessment (detection bias)	Low risk	Self-reporting.<br><br>"One a participant signed up, they were sent an automated email containing a link to the baseline survey and a unique username and pasword."<br><br>"Data were colleted online immediately after completion of the I-DICIDE or control website , and at 6 and 12 motnhs. An electronic participant database automatically sent women email promts at 6 and months with a link to the corresponding version of the weibsite. Women were asked to log in again with their existing username and password to complete their survey questions"
Incomplete outcome data (attrition bias)	Low risk	"Data analyses were done according to intention-to-treat principles, accounting for missing data, and adjusted for outcome data score."<br><br>"Follow-up response rates were higher than anticpated, and similar across groups. In the intervention group 7% complated immediate follow-up, 80% compalted 6-months follow-up and 79% complated 12-month follow-up. In the control group, 83% compalted immediate follow-up, 85% completed 6-month follow-up, and 80% complated 12-months follow-up."
Selective reporting (reporting bias)	Low risk	Trial registered on ACTRN with the protocol that has been previously published. Results are available on all outcomes.

Koziol-McLain, 2018

Bias	Authors' judgement	Support for judgement
Random sequence generation (selection	Low risk	Fully automated Web-based two-arm parallel RCT.

bias)		"Computer-generated randomization was based on a minimization scheme with stratification by severity of violence and children."
Allocation concealment (selection bias)	Low risk	The participant's allocation was kept secret from herself and the study team in New Zealand. Allocation remained entirely concealed until all baseline information was obtained. There was no procedural difference between arms until after baseline measures were obtained.
Blinding of participants and personnel (performance bias)	Low risk	Both participants and personnel (RAs) were blinded to group assignments. The particpant's allocation was kept secret from herself and the study team in New Zealand.<br>In the published protocol article it stated that, "All New Zealand trial investigators and team members are blinded to group assignment, with the exception of the data manager (JC) and trial statistician (ACV), who are responsible for the production of data monitoring reports."
Blinding of outcome assessment (detection bias)	Low risk	Self-reportedo utcomes. Particpants completed assessments at aeach time perioed regardless of prior assessment completions.<br><br>"Plans fo all inferential analyses were finalized before allocation unblinding in a full statistical anlaysis plan."<br><br>"A blind review, absent any information regarding allocation, was undertaken for each outcome for an assessment of missingness, a visual assessment of residual normality, an assessment of the covariance structure of the repeated measures, and an assessment of candidate covariates."
Incomplete outcome data (attrition bias)	Low risk	Intention-to-treat; 75% completed all three follow-up assessments – 73% assigned to control group and 76% assigned to intervention group. A 35% attriation rate was expected.
Selective reporting (reporting bias)	Low risk	Trial registered on ANZCTR with the protocol that has been published. Results described on 2 primary outcomes and the remaining secondary outcomes are described in appendices.

McFarlane, 2002

Bias	Authors' judgement	Support for judgement
Random sequence generation (selection bias	Unclear risk	"Sampling with randomization to treatment or control group." Doesn't specify exactly the randomization was done.
Allocation concealment (selection bias)	Unclear risk	Not stated.
Blinding of participants and personnel (performance bias)	High risk	"The investigator that entered the woman into the study completed all follow-up telephone calls." Which means personnel was not blinded. Participants were introduced to the study beforehand, but it doesn't specify if they were blinded to receive intervention or not.
Blinding of outcome assessment (detection bias)	Unclear risk	Not stated.
Incomplete outcome data (attrition bias)	Low risk	"The rention rate was 100% for the intervention group and

		98.7% for the controls (the woman who committed suicide was in the control group).”
Selective reporting (reporting bias)	Unclear risk	Trial was not registered and no protocol available (author has been contacted).

Stevens, 2015

Bias	Authors' judgement	Support for judgement
Random sequence generation (selection bias	Low risk	“Research participants were required to complete a baseline assessment over the phone with a research assistant to be randomly assigned to an experimental condition.”; “Assignment to condition was based on a computer-generated random number table.”
Allocation concealment (selection bias)	Unclear risk	Not stated.
Blinding of participants and personnel (performance bias)	High risk	RA was randomly assigned to an experimental condition. “Numerous steps were taken to keep the research assistants unaware of study condition.” For example, 1) the interventionists made calls in separate offices from Ras, 2) RAs were not given files containing condition assignment until the end of data collection, 3) the interventionists called participants in both study conditions so that a participant's infrequent mention to a RA of a conversation with a study nurse would not automatically reveal study condition.”<br><br>Participants could not blinded due to the overt nature of intervention (phone calls).
Blinding of outcome assessment (detection bias)	High risk	“The independent evaluators completed the measures listed below with participants over the phone at baseline (pre-intervention), at 3 months (mid-intervention), and at 6 months (post-intervention).” Due to the overt nature of the intervention, participants were aware of their intervention when responding to the outcome questions.
Incomplete outcome data (attrition bias)	Low risk	“There was a 76% retention rate for the intervention group from baseline to 3 monhts, and a 77% retention rate for the control group from baseline to 3 months. There was a 70% retention rate for the intervention group a 6 months and a 72% retention rate for the control group at 6 months.”
Selective reporting (reporting bias)	Unclear	Trial has not been registered.

Zlotnick, 2018

Bias	Authors' judgement	Support for judgement
Random sequence generation (selection bias)	Low risk	“The (computer) narrator "flipped a coin" and participants were randomized into the control or intervention.”
Allocation concealment (selection bias)	Unclear risk	Not stated.
Blinding of participants and personnel (performance bias)	Unclear risk	Not stated.
Blinding of outcome assessment (detection bias)	Unclear risk	All assessments consisted of self-report measures, which were computer-delivered. However, as it is inclear of whether the participants were aware of their randomisation, it is also unclear wheather or not this has influenced their

		reports.
Incomplete outcome data (attrition bias)	Low risk	2 of 28 women were lost to follow-up in the intervention group; 2 of 25 women were lost to follow-up for the control group
Selective reporting (reporting bias)	High risk	Trial registered in ClinicalTrials.gov (NCT02370394). There is unreported data on three pre-specified outcomes.
