# Supplementary material for: Intimate Partner Violence and Electronic Health Interventions: Systematic Review and Meta-Analysis of Randomized Trials
Source: J Med Internet Res. 2020 Dec 11;22(12):e22361. doi: 10.2196/22361 (PMC7762681; doi:10.2196/22361)

Multimedia Appendix 4: Additional meta-analyses

Figure S1: Effect of eHealth versus no eHealth intervention on depression

Study or Subgroup	eHealth Mean	eHealth SD	eHealth Total	Control Mean	Control SD	Control Total	Weight	Std. Mean Difference IV, Random, 95% CI
Constantiono 2015	14.9	3.2	11	26.6	5.5	11	3.7%	−2.50 [−3.67, −1.33]
Glass 2017	26.82	22.75	337	26.73	22.82	335	26.4%	0.00 [−0.15, 0.16]
Hegarty 2019	21.9	19.3	165	21.5	19.3	132	23.3%	0.02 [−0.21, 0.25]
Koziol−McLain 2018	22.59	21.38	172	23.3	20.48	184	24.1%	−0.03 [−0.24, 0.17]
Stevens 2015	17.7	14.3	129	19.8	12.3	124	22.5%	−0.16 [−0.40, 0.09]
**Total (95% CI)**			**814**			**786**	**100.0%**	**−0.13 [−0.37, 0.11]**

Heterogeneity: Tau^2 = 0.05; Chi^2 = 18.51, df = 4 (P = 0.0010); I^2 = 78%
Test for overall effect: Z = 1.06 (P = 0.29)

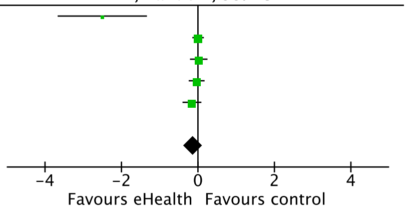

Figure S2. Effect of eHealth versus no eHealth on depression: Sensitivity analysis: outlier excluded [CESD scale]

Study or Subgroup	eHealth Mean	eHealth SD	eHealth Total	Control Mean	Control SD	Control Total	Weight	Mean Difference IV, Random, 95% CI
Glass 2017	26.82	22.75	337	26.73	22.82	335	30.0%	0.09 [−3.36, 3.54]
Hegarty 2019	21.9	19.3	165	21.5	19.3	132	18.2%	0.40 [−4.02, 4.82]
Koziol−McLain 2018	22.59	21.38	172	23.3	20.48	184	18.8%	−0.71 [−5.06, 3.64]
Stevens 2015	17.7	14.3	129	19.8	12.3	124	33.0%	−2.10 [−5.38, 1.18]
**Total (95% CI)**			**803**			**775**	**100.0%**	**−0.73 [−2.61, 1.16]**

Heterogeneity: Tau^2 = 0.00; Chi^2 = 1.14, df = 3 (P = 0.77); I^2 = 0%
Test for overall effect: Z = 0.76 (P = 0.45)

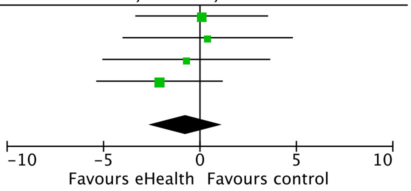

Figure S3. Effect of eHealth versus no eHealth on PTSD

Study or Subgroup	eHealth Mean	eHealth SD	eHealth Total	Control Mean	Control SD	Control Total	Weight	Mean Difference IV, Random, 95% CI
Glass 2017	15.83	6.49	337	16.06	6.61	335	88.2%	−0.23 [−1.22, 0.76]
Koziol−McLain 2018	39.48	17.07	175	38.35	16.28	167	6.9%	1.13 [−2.40, 4.66]
Stevens 2015	36.8	18.4	129	36.5	15.6	124	4.9%	0.30 [−3.90, 4.50]
**Total (95% CI)**			**641**			**626**	**100.0%**	**−0.11 [−1.04, 0.82]**

Heterogeneity: Tau^2 = 0.00; Chi^2 = 0.57, df = 2 (P = 0.75); I^2 = 0%
Test for overall effect: Z = 0.23 (P = 0.82)

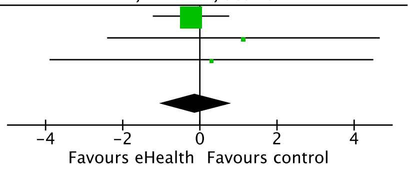

Figure S4: Effect of eHealth versus no eHealth on overall IPV: Subgroup analysis: low risk versus high risk of bias trials

	eHealth			Control				Std. Mean Difference
Study or Subgroup	Mean	SD	Total	Mean	SD	Total	Weight	IV, Random, 95% CI
**1.8.1 Low risk**								
Glass 2017	38.98	16.96	337	39.33	16.88	335	40.4%	−0.02 [−0.17, 0.13]
Koziol-McLain 2018	36.04	16.97	173	36.64	16.41	184	21.4%	−0.04 [−0.24, 0.17]
**Subtotal (95% CI)**			**510**			**519**	**61.8%**	**−0.03 [−0.15, 0.10]**
Heterogeneity: Tau² = 0.00; Chi² = 0.01, df = 1 (P = 0.91); I² = 0%								
Test for overall effect: Z = 0.42 (P = 0.68)								
**1.8.2 High risk**								
Hegarty 2019	17.1	20.5	178	17	19.5	155	19.9%	0.00 [−0.21, 0.22]
Stevens 2015	9.8	21.8	129	8.7	13.7	124	15.2%	0.06 [−0.19, 0.31]
Zlotnick 2018	12.4	12.4	28	16.5	20.2	25	3.1%	−0.24 [−0.79, 0.30]
**Subtotal (95% CI)**			**335**			**304**	**38.2%**	**0.01 [−0.15, 0.16]**
Heterogeneity: Tau² = 0.00; Chi² = 1.00, df = 2 (P = 0.61); I² = 0%								
Test for overall effect: Z = 0.08 (P = 0.94)								
**Total (95% CI)**			**845**			**823**	**100.0%**	**−0.01 [−0.11, 0.08]**
Heterogeneity: Tau² = 0.00; Chi² = 1.12, df = 4 (P = 0.89); I² = 0%								
Test for overall effect: Z = 0.28 (P = 0.78)								
Test for subgroup differences: Chi² = 0.10, df = 1 (P = 0.75), I² = 0%								

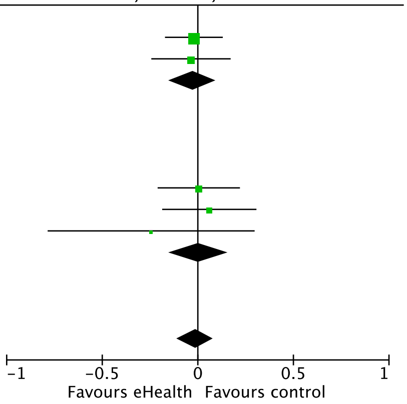

Figure S5: Effect of eHealth versus no eHealth intervention on overall IPV: Subgroup analysis: type of scale

	eHealth			Control				Std. Mean Difference
Study or Subgroup	Mean	SD	Total	Mean	SD	Total	Weight	IV, Random, 95% CI
**1.5.1 WEB**								
Koziol-McLain 2018	36.04	16.97	173	36.64	16.41	184	21.4%	−0.04 [−0.24, 0.17]
Glass 2017	38.98	16.96	337	39.33	16.88	335	40.4%	−0.02 [−0.17, 0.13]
**Subtotal (95% CI)**			**510**			**519**	**61.8%**	**−0.03 [−0.15, 0.10]**
Heterogeneity: Tau² = 0.00; Chi² = 0.01, df = 1 (P = 0.91); I² = 0%								
Test for overall effect: Z = 0.42 (P = 0.68)								
**1.5.2 CAS**								
Zlotnick 2018	12.4	12.4	28	16.5	20.2	25	3.1%	−0.24 [−0.79, 0.30]
Hegarty 2019	17.1	20.5	178	17	19.5	155	19.9%	0.00 [−0.21, 0.22]
Stevens 2015	9.8	21.8	129	8.7	13.7	124	15.2%	0.06 [−0.19, 0.31]
**Subtotal (95% CI)**			**335**			**304**	**38.2%**	**0.01 [−0.15, 0.16]**
Heterogeneity: Tau² = 0.00; Chi² = 1.00, df = 2 (P = 0.61); I² = 0%								
Test for overall effect: Z = 0.08 (P = 0.94)								
**Total (95% CI)**			**845**			**823**	**100.0%**	**−0.01 [−0.11, 0.08]**
Heterogeneity: Tau² = 0.00; Chi² = 1.12, df = 4 (P = 0.89); I² = 0%								
Test for overall effect: Z = 0.28 (P = 0.78)								
Test for subgroup differences: Chi² = 0.10, df = 1 (P = 0.75), I² = 0%								

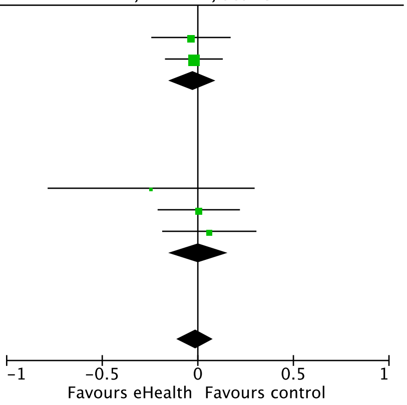

Figure S6: Effect of eHealth versus no eHealth intervention on overall IPV: Subgroup analysis: type of eHealth intervention

	eHealth			Control				Std. Mean Difference
**Study or Subgroup**	**Mean**	**SD**	**Total**	**Mean**	**SD**	**Total**	**Weight**	**IV, Random, 95% CI**
**1.6.1 Telephone support**								
Stevens 2015	9.8	21.8	129	8.7	13.7	124	15.2%	0.06 [−0.19, 0.31]
**Subtotal (95% CI)**			**129**			**124**	**15.2%**	**0.06 [−0.19, 0.31]**
Heterogeneity: Not applicable								
Test for overall effect: Z = 0.48 (P = 0.63)								
**1.6.2 Online safety decision aid**								
Glass 2017	38.98	16.96	337	39.33	16.88	335	40.4%	−0.02 [−0.17, 0.13]
Hegarty 2019	17.1	20.5	178	17	19.5	155	19.9%	0.00 [−0.21, 0.22]
Koziol-McLain 2018	36.04	16.97	173	36.64	16.41	184	21.4%	−0.04 [−0.24, 0.17]
**Subtotal (95% CI)**			**688**			**674**	**81.7%**	**−0.02 [−0.12, 0.09]**
Heterogeneity: $Tau^2 = 0.00$; $Chi^2 = 0.07$, df = 2 (P = 0.96); $I^2 = 0\%$								
Test for overall effect: Z = 0.34 (P = 0.73)								
**1.6.3 Online education**								
Zlotnick 2018	12.4	12.4	28	16.5	20.2	25	3.1%	−0.24 [−0.79, 0.30]
**Subtotal (95% CI)**			**28**			**25**	**3.1%**	**−0.24 [−0.79, 0.30]**
Heterogeneity: Not applicable								
Test for overall effect: Z = 0.88 (P = 0.38)								
**Total (95% CI)**			**845**			**823**	**100.0%**	**−0.01 [−0.11, 0.08]**
Heterogeneity: $Tau^2 = 0.00$; $Chi^2 = 1.12$, df = 4 (P = 0.89); $I^2 = 0\%$								
Test for overall effect: Z = 0.28 (P = 0.78)								
Test for subgroup differences: $Chi^2 = 1.05$, df = 2 (P = 0.59), $I^2 = 0\%$								

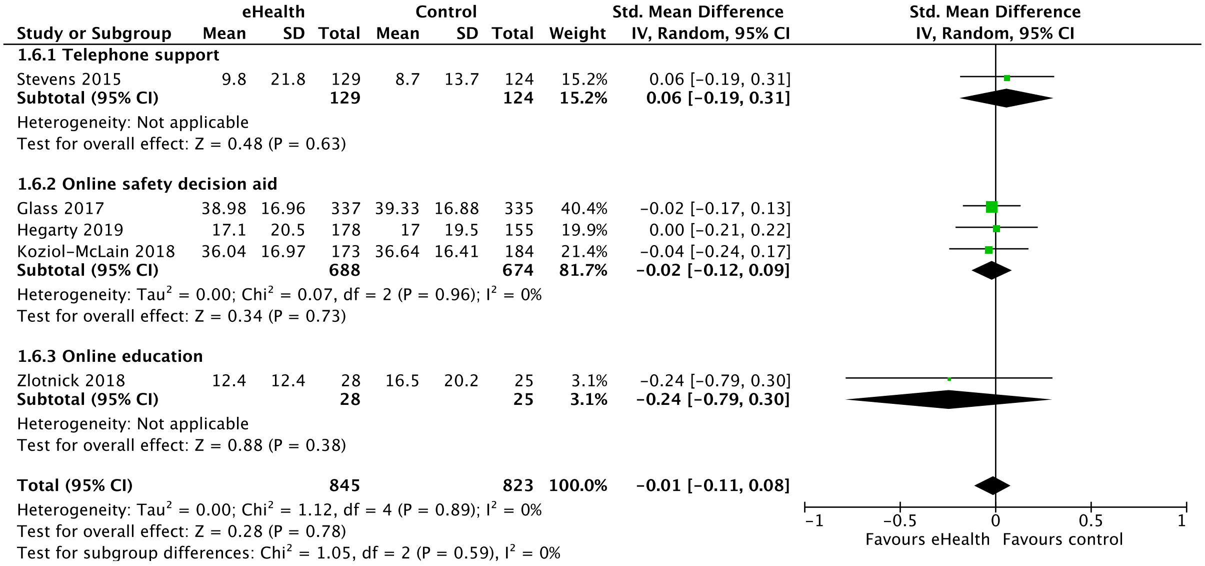

Supplement: Multimedia Appendix 4 [file jmir_v22i12e22361_app4.pdf]
